# Hantavirus in African Wood Mouse, Guinea

**DOI:** 10.3201/eid1205.051487

**Published:** 2006-05

**Authors:** Boris Klempa, Elisabeth Fichet-Calvet, Emilie Lecompte, Brita Auste, Vladimir Aniskin, Helga Meisel, Christiane Denys, Lamine Koivogui, Jan ter Meulen, Detlev H. Krüger

**Affiliations:** * Charité Medical School, Berlin, Germany;; † Slovak Academy of Sciences, Bratislava, Slovak Republic;; ‡ Museum National d'Histoire Naturelle, Paris, France;; § Philipps University, Marburg, Germany;; ¶ Severtsov Institute of Ecology and Evolution, Moscow, Russia;; # Viral Hemorrhagic Fever Project, Conakry, Guinea

**Keywords:** Hantavirus, Africa, RNA sequence analysis, dispatch

## Abstract

Hantaviruses are rodentborne, emerging viruses that cause life-threatening human diseases in Eurasia and the Americas. We detected hantavirus genome sequences in an African wood mouse (*Hylomyscus simus*) captured in Sangassou, Guinea. Sequence and phylogenetic analyses of the genetic material demonstrate a novel hantavirus species, which we propose to name "Sangassou virus."

Hantaviruses, family *Bunyaviridae*, are emerging viruses that cause 2 life-threatening human zoonoses: hemorrhagic fever with renal syndrome (HFRS) and hantavirus pulmonary syndrome (HPS, also known as hantavirus cardiopulmonary syndrome). The virus genome consists of 3 segments of negative-stranded RNA; the large (L) segment encodes viral RNA-dependent RNA polymerase, the medium (M) segment encodes glycoprotein precursor, and the small (S) segment encodes nucleocapsid protein. In contrast to other members of the *Bunyaviridae*, hantaviruses are not transmitted by arthropods but are spread by aerosolized excreta of rodents of the family *Muridae*, their natural hosts (*1**–**4*).

Hantaviruses have a strong association with certain reservoir host species. Phylogenetic analyses have divided hantaviruses into 3 major groups according to 3 subfamilies of their natural hosts. Hantaan virus (HTNV), Seoul virus (SEOV), and Dobrava virus (DOBV), which cause HFRS in Asia and Europe, are examples of *Murinae*–associated viruses. Puumala virus (PUUV), which causes a mild form of HFRS in Europe, and the less pathogenic Tula virus (TULV) are *Arvicolinae*–associated hantaviruses. In 1993, Sin Nombre virus (SNV) was discovered in the United States as the first member of the third group, *Sigmodontinae*–associated hantaviruses. SNV from North America and Andes virus (ANDV) from South America are the most prominent examples of viruses causing HPS (*3**,**5*).

Hantaviruses cause human diseases predominantly in Asia, Europe, and the Americas. Few studies have considered hantaviruses in Africa; such reports originated from serologic surveys of human populations. In this study, we report detection and initial genetic characterization of the first indigenous African hantavirus detected in an African wood mouse (*Hylomyscus simus*) in Sangassou, Guinea.

## The Study

In a survey for rodentborne hemorrhagic fever viruses, 612 small rodents representing 17 different genera (most abundant were *Mastomys* [n = 325], *Praomys* [n = 95], and *Nannomys* [n = 83]) were trapped in Guinea from 2002 to 2004 and screened for hantavirus RNA by reverse transcription–polymerase chain reaction (RT-PCR). We used a molecular genetic approach to screen the rodent population because hantavirus RNA (as shown for SNV) can be amplified from the blood of persistently infected mice by RT-PCR over a long period (*6*). For this purpose, we developed a nested RT-PCR assay to detect currently known and possible novel members of the genus *Hantavirus*. The assay was based on degenerated primers (HAN-L-F1: 5´-ATGTAYGTBAGTGCWGATGC-3´ and HAN-L-R1: 5´-AACCADTCWGTYCCRTCATC-3´ for primary PCR, HAN-L-F2: 5´-TGCWGATGCHACIAARTGGTC-3´ and HAN-L-R2: 5´-GCRTCRTCWGARTGRTGDGCAA-3´ for nested PCR) designed from an alignment of all available nucleotide sequences of the highly conserved L segment. For the RT-PCR, total RNA was extracted from wild-trapped rodent blood (preserved in liquid nitrogen) with the Blood RNA kit (Peqlab, Erlangen, Germany) and reverse transcribed with random hexamers as primers.

A sample (designated SA14) obtained from 1 of 4 investigated African wood mice (*H*. *simus*) generated an L segment–derived PCR product of expected size. This rodent was trapped in January 2004 in a forest habitat near the village of Sangassou, near Macenta, Guinea (8°36´49´´N, 9°28´27´´W). Its karyotype was determined (2n = 48, fundamental no. = 74, autosomal fundamental no. = 70) and the complete cytochrome *b* gene was sequenced and compared with the genes of other *Hylomyscus* species recognized in the most recent revision of the genus (*7*) (GenBank accession nos. DQ212188 and DQ078229-DQ078245).

The 412-nucleotide (nt) sequence of the first PCR product was determined by amplification, cloning, and sequencing of overlapping fragments generated by 2 seminested PCRs (GenBank accession no. DQ268652). Additional S and M segment–specific nested PCR assays were developed to further characterize the novel virus. PCR fragments of 837 nt and 694 nt could be analyzed (GenBank accession nos. DQ268650 and DQ268651, respectively).

The Table shows nucleotide sequence identity comparisons between SA14 and other members of the genus *Hantavirus*. *Murinae*-associated hantaviruses (HTNV, DOBV, SEOV) showed the highest similarity to the SA14 sequence in all 3 genomic segments (71.3%–77.1% for S, 72.9%–77.9% for M, and 72.3%–75.9% for L). This similarity is consistent with the evolutionary relationship of their putative hosts. On the amino acid level, corresponding sequences of deduced viral proteins showed highest similarity with those of other *Murinae*-associated hantaviruses (81.7%–88.5% for S, 82.2%–89.6% for M, and 85.4%–87.5% for L). The amino acid sequence divergence between SA14 and most related hantaviruses corresponds to that typically found between different virus species, e.g., SNV and ANDV.

**Table:** Similarity of (% Identity with SA14 partial sequences) SA14 partial S, M, and L segment sequences with those of other hantaviruses*†

Hantavirus	S segment		M segment		L segment	
	nt	aa	nt	aa	nt	aa
HTNV_76-118_	71.3	81.7	77.9	89.6	72.3	86.1
SEOV_80-39_	75.8	82.4	72.9	82.2	75.9	87.5
DOBV_SK/Aa_	77.1	88.5	77.6	89.6	73.0	85.4
PUUV_CG1820_	61.4	61.1	62.1	61.4	69.6	72.2
TULV_Moravia_	62.0	62.3	62.5	62.7	65.2	72.2
SNV_NM H10_	63.2	62.3	60.8	62.3	68.9	72.2
ANDV_Chile-R123_	62.0	62.7	65.5	62.7	68.6	72.9

*S, small; M, medium; L, large; HTNV, Hantaan virus; SEOV, Seoul virus; DOBV, Dobrava virus; PUUV, Puumala virus; TULV, Tula virus; SNV, Sin Nombre virus; ANDV, Andes virus. †837 nucleotides (nt) of the S segment (positions 394–1230), 694 nt of the M segment (positions 2281–2974), and 412 nt of the L segment (positions 2956–3367) and the deduced amino acid (aa) sequences (279 aa, position 120–398 of the nucleocapsid protein; 231 aa, positions 748–978 of the glycoprotein precursor; 137 aa, positions 974–1110 of the viral RNA-dependent RNA polymerase) have been compared. Fragment positions were defined according to complete sequences of HTNV strain 76-118 (GenBank accession nos. NC_005218, NC_005219, and NC_005222).

The S-, M-, and L-segment–derived nucleotide sequences of SA14 were subjected to maximum likelihood (ML) and neighbor-joining (NJ) phylogenetic analyses with available nucleotide sequences of other *Murinae*-associated hantaviruses. PUUV, TULV, SNV, and ANDV sequences were used as outgroups. In the S segment ML phylogenetic tree (Figure), SA14 clustered with other *Murinae*-associated viruses. As expected, 3 clades were formed by members of the 3 established hantavirus species (HTNV, DOBV, and SEOV). Within this well-supported cluster, the SA14 sequence is most closely related to the DOBV clade. The M segment analysis showed an identical placement of SA14 with strong statistical support (PUZZLE [8] and bootstrap values above the threshold value of 70%, data not shown). In L-segment phylogeny, the resolution of the tree was decreased. The SA14 L sequence did not join with statistical support any of the 3 groups but formed a fourth clade within the cluster of *Murinae*-associated hantaviruses. (PUZZLE and bootstrap values above the threshold for the placement of SA14 within the *Murinae*-associated viruses but <50% in both analyses for its clustering with any of these viruses, data not shown).

**Figure:**
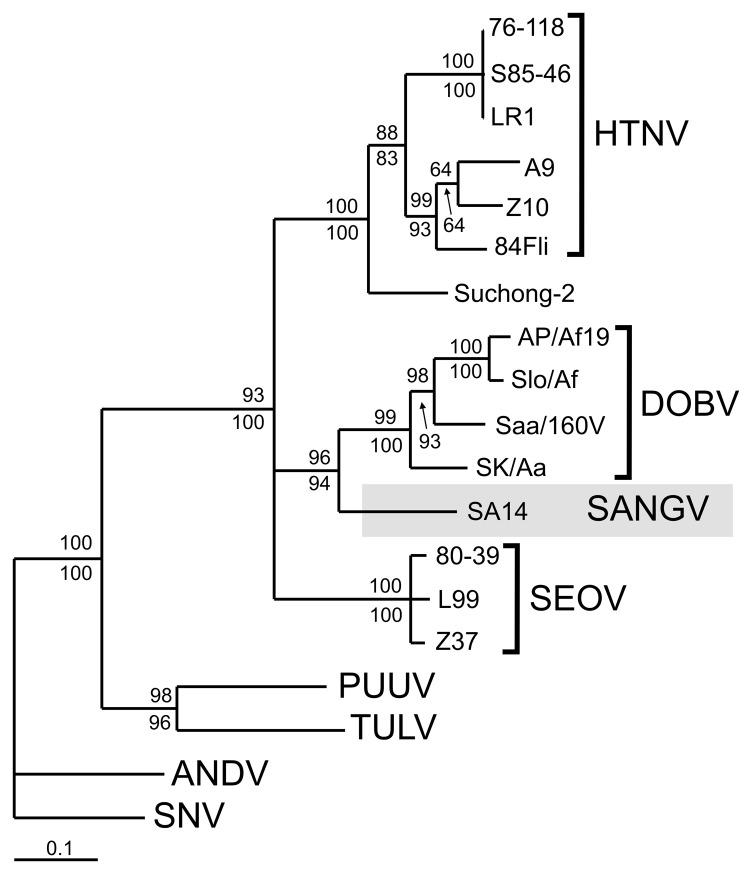
Maximum likelihood phylogenetic tree of hantaviruses showing the placement of SA14 (Sangassou virus [SANGV], indicated by gray shading). Partial S segment genome sequences (837 nucleotides, positions 394–1230) were used to calculate the tree with TREE-PUZZLE program (*8*). The Tamura-Nei evolutionary model was used; the values above the branches represent PUZZLE support values. The values below the branches represent bootstrap values of the corresponding neighbor-joining tree computed with PAUP* program (*9*) using 10,000 bootstrap replicates. The scale bar indicates an evolutionary distance of 0.1 nucleotide substitutions per position in the sequence. HTNV, Hantaan virus; DOBV, Dobrava virus; SEOV, Seoul virus; PUUV, Puumala virus; TULV, Tula virus; ANDV, Andes virus; SNV, Sin Nombre virus.

## Conclusions

Extended fragments of novel hantavirus S, M, and L genome segments were recovered from an arboreal African rodent. They clearly represent genetic material of a novel hantavirus species because their amino acid sequence is significantly (≈15%) divergent from those of other hantaviruses, they form a distinct clade in phylogenetic trees, and they were detected in a rodent species previously not recognized as a natural host of hantaviruses. We propose to name the new species Sangassou virus (SANGV) after the locality where it was detected.

Although hantaviruses are emerging viruses circulating in Asia, Europe, and the Americas, our study represents the first genetic evidence for hantaviruses in Africa. Suspected human hantavirus infections have been reported in various African countries (*10**–**13*). Most of these are seroepidemiologic studies reporting antibodies reacting with HTNV antigen. However, *Apodemus agrarius*, the natural host of HTNV, is not found in Africa. Based on the putative cross-reactivity of antigens from HTNV, SANGV, and other *Murinae*-associated viruses, human infections, at least in tropical forest parts of Africa where *Hylomyscus* species are prevalent, could be caused by SANGV or other *Murine*-associated hantaviruses.

To our knowledge, 1 case of HFRS has been reported in central Africa (*14*). Although HFRS is not a known disease in West or central Africa, one cannot ignore the potential pathogenicity of SANGV or other African hantaviruses. HFRS may be confused with other severe diseases (leptospirosis, rickettsiosis, other viral hemorrhagic fevers, plague, severe pneumonia, sepsis) or may be unrecognized because of poor health care. One should remember that HPS and *Sigmodontinae*-associated hantaviruses were not recognized until 1993, even in such a highly developed country as the United States.

Further studies are needed to verify the presence and distribution of hantaviruses in Africa and their potential impact on human health. These studies should focus on areas with forest activities, such as logging, which may bring humans into contact with viral reservoirs (*15*). Our data justify inclusion of hantavirus infection in the differential diagnosis of patients from Africa with unexplained febrile nephropathies or noncardiogenic pulmonary edema.
